# Serosurvey of Dogs for Human, Livestock, and Wildlife Pathogens, Uganda

**DOI:** 10.3201/eid1904.121143

**Published:** 2013-04

**Authors:** Javier Millán, Andrea D. Chirife, Gladys Kalema-Zikusoka, Oscar Cabezón, Jesús Muro, Ignasi Marco, Florence Cliquet, Luis León-Vizcaíno, Marine Wasniewski, Sonia Almería, Lawrence Mugisha

**Affiliations:** Universitat Autònoma de Barcelona, Bellaterra, Spain (J. Millán, A.D. Chirife, O. Cabezón, I. Marco, S. Almería);; Conservation through Public Health, Kampala, Uganda (G. Kalema-Zikusoka);; Ministry of Agriculture, Andorra la Vella, Andorra (J. Muro);; Nancy Laboratory for Rabies and Wildlife, Malzéville, France (F. Cliquet, M. Wasniewski);; University of Murcia, Murcia, Spain (L. León-Vizcaíno);; Centre de Recerca en Sanitat Animal, Bellaterra, Spain (S. Almería);; Makerere University, Kampala (L. Mugisha);; Conservation and Ecosystem Health Alliance, Kampala (L. Mugisha)

**Keywords:** zoonoses, dogs, livestock, domestic animals, wildlife, pathogens, serologic surveys, preventive strategies, rabies, visceral leishmaniasis, Toxoplasma gondii, viruses, parasites, bacteria

**To the Editor:** Domestic dogs live in close association with humans and livestock, participating in the transmission of diseases of zoonotic, veterinary, and conservation interest (*1*,*2*). Most households in Uganda have traditionally kept dogs for hunting and for help with herding, security, and guarding livestock. Most dogs receive no prophylactic measures (e.g., vaccinations) and roam freely; this situation exposes them to pathogens from eating garbage, rodents, and stillborn animals and other carcasses and through inhalation during scent communication. Thus, dogs are a reservoir for certain pathogens and a useful sentinel for others (*3*).

In 2011, serum samples were obtained from 116 mixed-breed dogs during a rabies vaccination campaign in and near 3 national parks in southwestern Uganda; the dogs were >4 months of age and were voluntarily brought in by their owners (Figure, Appendix, Table). Two of the parks, Bwindi Impenetrable (BI) and Mgahinga Gorilla (MG), have some of the most biologically diverse tropical forests in eastern Africa and are home to mountain gorillas. The third park, Queen Elizabeth (QE), is home to populations of protected carnivores and ungulates. The parks lie within a densely populated rural landscape; in some areas, the population is as high as 500 persons/km^2^. 

**Figure F1:**
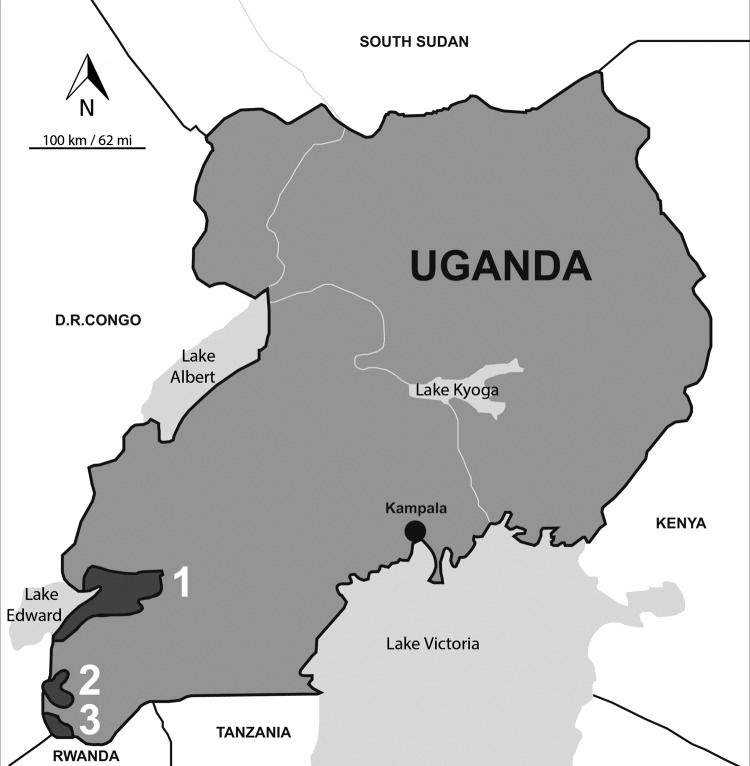
Map of Uganda showing 3 areas where a serosurvery for human and animal pathogens was conducted among dogs. 1, Queen Elizabeth National Park; 2, Bwindi Impenetrable National Park; 3, Mgahinga Gorilla National Park.

**Table:** Methodology and seroprevalence for selected pathogens in rural dogs in 3 national parks, Uganda, 2011*

Pathogen	Test, cutoff value, and ref or kit	National park										
		All 3 parks			Queen Elizabeth†			Bwindi Impenetrable‡			Mgahinga Gorilla§	
		Sample size	Prevalence, % (95% CI)		Sample size	Prevalence, % (95% CI)		Sample size	Prevalence, % (95% CI)		Sample size	Prevalence, % (95% CI)
Rabies virus¶	FAVN, 0.24 IU/mL (*4*)	101	19.8 (12.7–28.6)		23	21.7 (9.0–43.3)		56	19.6 (11.0–32.0)		22	16.7 (5.9–37.2)
CDV	c-ELISA, Ingezim Moquillo IgG#	92	100.0 (95.9–100)		30	100 (88.8–100.0)		39	100 (91.4–100.0)		23	100 (85.4–100.0)
CPV	c-ELISA, Ingezim CPV#	92	65.2 (54.9–74.5)		26	80.8 (61.7–92.1)		43	76.7 (61.7–87.6)		23	26.1 (12.0–47.8)
Leptospira interrogans**	MAT, 1:200 (*15*)	105	26.7 (19.0–36.1)		27	25.9 (12.4–46.2)		55	29.1 (17.9–42.7)		23	21.7 (9.0–43.3)
Leishmania sp.††	c-ELISA, Ingezim Leishmania#	92	19.6 (12.3–29.2)		26	19.2 (7.9–38.3)		43	25.6 (14.6–40.6)		23	8.7 (1.6–27.8)
Toxoplasma gondii	MAT, 1:25 (*3*)	109	90.8 (83.6–95.1)		30	90.0 (73.7–97.2)		56	98.2 (90.5–99.9)		23	73.9 (52.2–88.0)
Neospora caninum	c-ELISA, 30% (*3*)	109	27.5 (19.6–36.6)		30	26.7 (13.1–45.0)		56	32.1 (21.2–45.5)		23	30.4 (14.5–52.2)

*Ref, reference; FAVN, fluorescent antibody virus neutralization; CPV, canine parvovirus; c-ELISA, competitive ELISA; CDV, canine distemper virus; MAT, modified agglutination test. †0°12′ S, 30°0′ E (savannah). ‡1°0′ S, 29°42′ E (tropical forest). §1°16′ S, 29°40′ E (tropical forest). ¶Four dogs vaccinated against rabies in Queen Elizabeth are not included in these results. #Manufactured by Ingenasa, Madrid, Spain. **Fourteen serovars were investigated. Of the dogs seropositive, 71.5% were seropositive to 1 serovar and 28.5% to 2 serovars. Reacting serovars were Icterohaemorragiae (42.8% of positive dogs), Canicola (39.2%), Pyrogenes (21.4%), Tarassovi (10.7%), and Gryppothiposa and Australis (7.2% each). ††Antibodies probably correspond to contact with *Leishmania donovani*.

Of the 116 sampled dogs, 4 had been vaccinated against rabies by the authors in 2010 in QE (not included in rabies results), and 11 (all males) had been castrated by local animal healers before serum samples were obtained. The samples were used to test for seroprevalence rates to rabies virus (RABV), canine distemper virus (CDV), canine parvovirus (CPV), *Leptospira interrogans*, *Leishmania* sp., *Toxoplasma gondii*, and *Neospora caninum* (Table). Seroprevalence rates ranged from 20% to 100% (Table). CPV seroprevalence was higher in BI and QE than in MG (χ^2^ >12.6, p<0.001); *T. gondii* seroprevalence was higher in BI than in MG (Fisher p = 0.002); and RABV seroprevalence was higher in castrated than noncastrated dogs (50% vs. 10%; Fisher p = 0.005).

For humans, the domestic dog is the main source of exposure to RABV. The possibility that the presence of the rabies titers in the dog serum samples was due to a previous vaccination can be ruled out because the only previous recent campaign in the area was conducted by the authors. Antibodies against RABV in apparently healthy dogs have been reported in Africa (*6*), and rabies seems to be not invariably fatal in dogs. Dogs that have recovered from a rabies infection are prone to shed RABV in their saliva for long periods (*7*). Antibodies against RABV were more frequently found in castrated dogs. This finding may be due to an increase in virus-related deaths among noncastrated dogs; such dogs tend to be more aggressive and to roam, so they may come more frequently into contact with pathogenic RABV strains.

Results indicate that both CDV and CPV are actively circulating in the studied dog populations. High CDV seroprevalence rates have been reported among other rural dog populations in Africa (*8*). Sick, debilitated pups are at high risk for predation by wild carnivores, so spillover may take place. A dog population exhibiting similar characteristics to the population we studied was believed to be the origin of the 1994 CDV epidemic among Serengeti wildlife (*8*). Furthermore, carnivores use feces for scent communication, so the probability of infection by CPV in wild carnivores in the study area may also be high.

In developing countries, leptospirosis is emerging as a major public health problem and also causes enormous economic losses because of disease in livestock (*9*). The most commonly detected serovars in this study were those that have rats and dogs as reservoirs (Table[Fig F1]). Visceral leishmaniasis in humans is also a major health problem in several areas of eastern Africa, where the number of cases has dramatically increased during the past 20 years. Transmission of *Leishmania donovani* in eastern Africa may take place through anthroponotic or zoonotic cycles, although, to our knowledge, no reservoir host had been identified (*10*).

The mean *T. gondii* seroprevalence detected during this survey appears to be the highest reported for dogs worldwide. This protozoon has implications for human and animal health, and dogs, who probably become infected with *T. gondii* when eating raw meat, are a good sentinel for environmental contamination by this parasite. On the other hand, dogs serve as the definitive host for *N. caninum*, which is a major cause of abortions in cattle and causes economic losses wherever it is enzootic.

Some of these diseases may also have implications for the conservation of endangered mountain gorillas. Diseases such as leptospirosis, toxoplasmosis, and especially, rabies could be fatal for gorillas, and there are unpublished reports of fights between hunting dogs and gorillas.

Our work should serve as a first step toward the establishment of preventive strategies for improvements in the health of humans and domestic animals living in rural Uganda and for the health of the country’s unique wildlife. Tracing the role of dogs in the cycle of the studied pathogens is crucial for the design of control programs.
